# Highly connected, non-redundant microRNA functional control in breast cancer molecular subtypes

**DOI:** 10.1098/rsfs.2020.0073

**Published:** 2021-06-11

**Authors:** Guillermo de Anda-Jáuregui, Jesús Espinal-Enríquez, Enrique Hernández-Lemus

**Affiliations:** ^1^ Computational Genomics, Instituto Nacional de Medicina Genómica, Mexico City, Mexico; ^2^ Cátedras CONACYT for Young Researchers, Consejo Nacional de Ciencia y Tecnología, Mexico City, Mexico; ^3^ Center for Complexity Sciences, Universidad Nacional Autónoma de México, Mexico City, Mexico

**Keywords:** micro-RNA, biological networks, hallmarks of cancer, computational oncology, multi-omics

## Abstract

Breast cancer is a complex, heterogeneous disease at the phenotypic and molecular level. In particular, the transcriptional regulatory programs are known to be significantly affected and such transcriptional alterations are able to capture some of the heterogeneity of the disease, leading to the emergence of breast cancer molecular subtypes. Recently, it has been found that network biology approaches to decipher such abnormal gene regulation programs, for instance by means of gene co-expression networks, have been able to recapitulate the differences between breast cancer subtypes providing elements to further understand their functional origins and consequences. Network biology approaches may be extended to include other co-expression patterns, like those found between genes and non-coding transcripts such as microRNAs (miRs). As is known, miRs play relevant roles in the establishment of normal and anomalous transcription processes. Commodore miRs (cdre-miRs) have been defined as miRs that, based on their connectivity and redundancy in co-expression networks, are potential control elements of biological functions. In this work, we reconstructed miR–gene co-expression networks for each breast cancer molecular subtype, from high throughput data in 424 samples from the Cancer Genome Atlas consortium. We identified cdre-miRs in three out of four molecular subtypes. We found that in each subtype, each cdre-miR was linked to a different set of associated genes, as well as a different set of associated biological functions. We used a systematic literature validation strategy, and identified that the associated biological functions to these cdre-miRs are *hallmarks of cancer* such as angiogenesis, cell adhesion, cell cycle and regulation of apoptosis. The relevance of such cdre-miRs as actionable molecular targets in breast cancer is still to be determined from functional studies.

## 1. Background

Breast cancer is a heterogeneous disease with many different manifestations. The heterogeneous nature of breast cancer can be observed at the transcriptional level, in the different gene expression patterns observed. These differences in breast cancer are at the basis of molecular classifications, such as the breast cancer molecular subtypes: Luminal A, Luminal B, Basal and HER2-enriched [1,2]. These different molecular patterns are associated with different physiopathological properties, which can be used for clinical applications [3,4].

The transcriptional patterns of breast cancer have been explored in previous works. Our group has found that, by representing the transcriptional program of breast cancer molecular subtypes as co-expression networks, it is possible to capture the differences found between each cancer manifestation [5]. We have also shown how genes with coordinated expression patterns are found associated with each cancer subtype, and through these, it is possible to identify and associate functional perturbations to molecular subtypes [6,7].

The regulatory programs of biological phenotypes are not limited to gene interactions. Elements such as non-coding RNAs are also involved in the regulation of gene expression. It has been shown that the transcriptional patterns of these non-coding RNAs also capture the heterogeneity of breast cancer molecular subtypes [8]. The species known as microRNA (miR) are a class of non-coding RNA that is currently a major study subject in cancer. Our group has studied such miRs from a network biology perspective [9].

Control in complex networks has important applications [10]. In the context of gene expression regulation, the control of gene expression, and more importantly, the concerted regulation of genes associated with biological functions, could have important biomedical applications. Similar concepts, such as master regulators [11–13], have been explored in different biological concepts, including cancer. In recent work, we introduced the concept of *Commodore miRs* (cdre-miRs): miRs that are highly connected and non-redundant in miR–gene co-expression networks in breast cancer, that are theoretically capable of controlling the state of specific biological functions by themselves [14]. In this work, we intend to explore whether this *Commodore* behaviour can be found for miRs in networks of different breast cancer subtypes, how these cdre-miRs differ in each subtype, and how they are potentially able to influence the activity of biological processes important for cancer manifestation.

## 2. Material and methods

**Figure 1.**
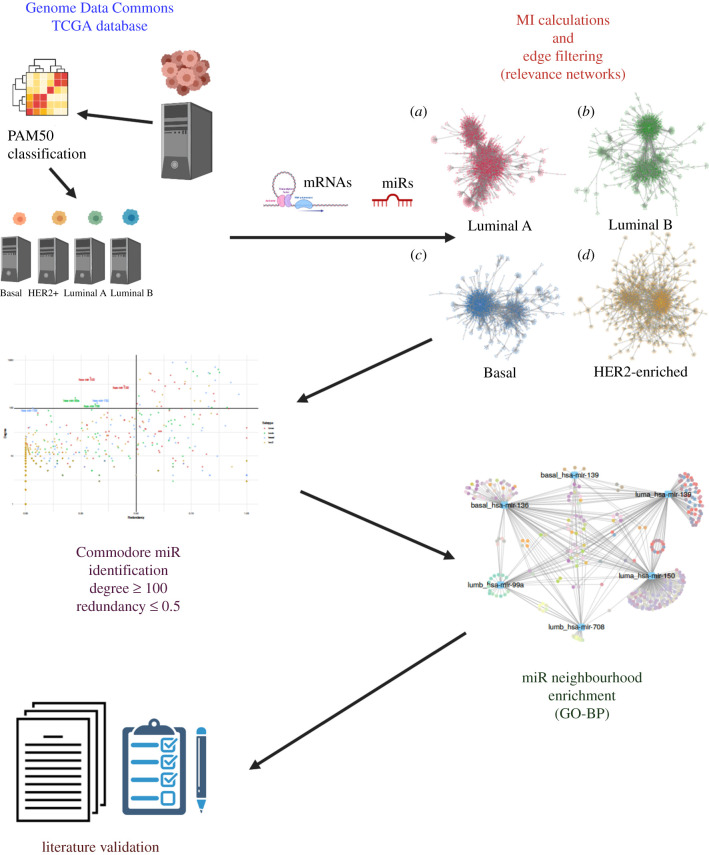
The cdre-miR analysis workflow.

The workflow that was followed in this paper consists of the breast cancer gene and miR data acquisition, the co-expression network reconstruction, the identification of cdre-miRs, the functional enrichment of cdre-miR neighbourhoods, and the literature validation of the identified biological functions. This workflow is represented in figure 1.

### 2.1. Expression data

Expression data for miR and genes in breast cancer were obtained from the Cancer Genome Atlas. The subset of breast cancer samples used in the 2012 TCGA publication [8] includes the molecular subtype sample classification. We acquired this information from the cBioportal website [15,16]. We downloaded the expression data for gene and miR, for each molecular subtype: Luminal A (lumA), Luminal B (lumB), Basal and HER2-enriched (HER2) from the Genome Data Commons website (https://portal.gdc.cancer.gov/repository). In total, 92 basal subtype samples, 57 HER2-enriched samples, 155 Luminal A samples and 120 Luminal B samples were acquired.

The datasets found in the GDC platform are processed according to the bioinformatic pipelines found in https://docs.gdc.cancer.gov/Data/Bioinformatics_Pipelines/Expression_mRNA_Pipeline/ for genes and https://docs.gdc.cancer.gov/Data/Bioinformatics_Pipelines/miRNA_Pipeline/ for miR, which are referenced in the relevant original publications [8,17]. For this work, we used FPKM—normalized data as expression values for mRNA, and reads per million miRNA mapping (RPMMM) data as expression values for miR.

### 2.2. MicroRNA–gene bipartite network reconstruction

We reconstructed a bipartite network representing the co-expression between miR and genes in each molecular subtype. For this, we used mutual information (MI) as a measure of miR–gene co-expression. MI has been widely used for the reconstruction of co-expression networks [5,18–22]. In previous work by our group, we have successfully reconstructed miR–gene co-expression networks using this approach [9,14].

For each molecular subtype, we calculated MI for each miR–gene pair based on their expression levels in order to fill an incidence matrix. We then selected the miR–gene pairs that will be connected in the network based on their MI values. Those pairs with an MI value above a certain threshold were kept as links in the network, while those with an MI value below the threshold were discarded.

This strategy is the same that was used by our group in previous miR–gene co-expression network studies [9,14]. The MI threshold selected for each network was set to be that which allowed us to keep the 0.9999 upper quantile of all possible links; this is based on a heuristic described by our group previously [23]. This allows to recover networks that have a comparable number of edges for each molecular subtype.

### 2.3. Network analyses

The bipartite networks were analysed for basic network topological properties using the igraph package for R [24]. The calculation of bipartite network properties, including the redundancy coefficient as defined in [25], was computed using the NetworkX package [26] for Python.

### 2.4. Commodore microRNA identification

In our previous work regarding miR–gene co-expression networks [14], we defined the concept of a cdre-miR: a miR that has a high number of neighbours, but a low redundancy coefficient (as defined by [25]) in a miR–gene co-expression network. Briefly, the redundancy coefficient of a node in a bipartite network measures the contribution of said node to the connectivity of the opposite layer: if a highly redundant node is removed, nodes in the opposite layer will remain connected; whereas if a non-redundant node is removed, the paths connecting the nodes in the opposite layer may be lost. Consistently with the work cited above, we are considering a highly connected, non-redundant miR node to be, in the context of breast cancer, that which has a degree *k* ≥ 100, and a redundancy coefficient *rc* ≤ 0.5.

### 2.5. Functional enrichment of Commodore microRNA neighbourhoods

Each identified cdre-miR has, by definition, a neighbourhood of at least 100 genes. We identified biological functions that are associated with these neighbourhoods, and therefore to each cdre-miR. We performed this functional enrichment through an over-representation analysis, using the HTSanalyzer package for R [27]. We tested over-representation of the genesets encompassed in the gene ontology biological process (GO-BP) database [28,29]. We considered a significance threshold of *Adjusted* *p*-*value* ≤ 10^−3^ in the hypergeometric test.

### 2.6. Functional category aggregation

We decided to present all the GO-BP categories found to be significantly associated with each cdre-miR. However, it is possible to leverage the ontological nature of the GO-BP database to group GO-BP categories that are both functionally related and composed of similar gene sets. To do this, we used the Wang similarity score [30], which measures the similarity between GO terms.

We calculated this similarity score for the GO-BP enriched for each cdre-miR of each subtype (using the GoSemSim package [31]). Then, we used this as the basis for a hierarchical clustering method, with which we generated for each cdre-miR, ten sets of functionally similar GO-BPs. We then selected as a *representative* GO-BP for each group, the GO-BP that had the lowest *Adjusted*.*p*-*value* within the group. The intention behind this is to obtain a more interpretable set of potential functional targets of cdre-miRs.

### 2.7. Literature validation

We performed systematic queries to the Pubmed database to identify previously reported associations between the cdre-miRs and the functions identified in this work. To do so, we used the Rentrez package for R (https://github.com/ropensci/rentrez). For each subtype, for each cdre-miR, we performed a query of the form mir+Representative GO−BP considering each of the 10 function groups associated with each cdre-miR.

## 3. Results

### 3.1. MicroRNA-gene co-expression networks

**Figure 2.**
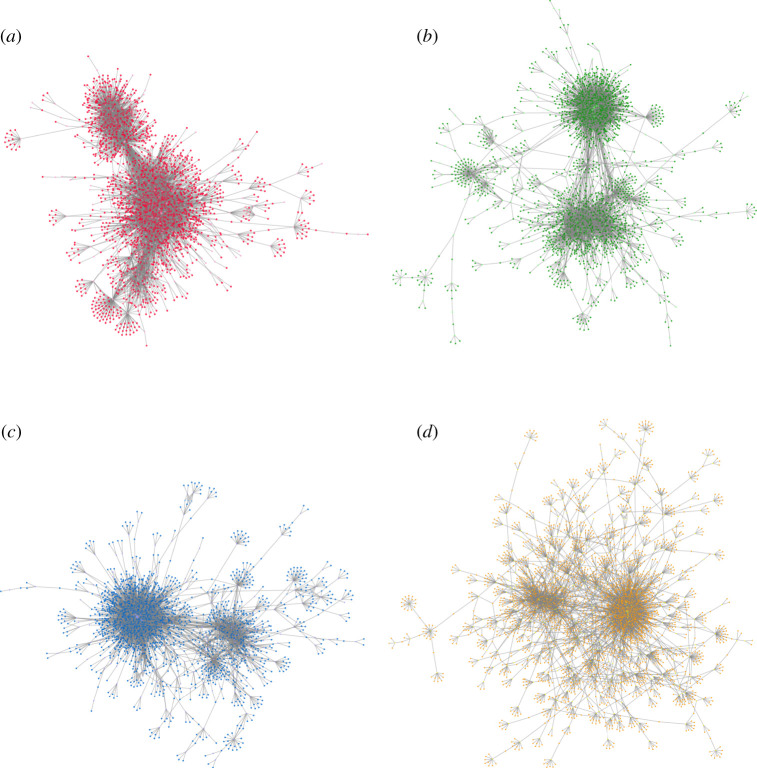
miR–gene co-expression network visualizations for each breast cancer molecular subtype; largest connected component shown. (*a*) Luminal A, (*b*) Luminal B, (*c*) Basal and (*d*) HER2-enriched.

We reconstructed miR–gene co-expression networks for each molecular subtype. These networks are comparable, by construction, in terms of the number of edges that they contain, and the number of miR and gene nodes (as they contain all the miRs and genes measured in the original experiments). The number of connected (*k* > 0) nodes and connected components (non-single nodes) in each network is variable, but they are, overall, comparable; this can be seen in table 1. Visualizations of the largest connected components are found in figure 2. Other network parameters, including degree distribution, are found in electronic supplementary material, file 2.

**Table 1.** Network parameters.

	Luminal A	Luminal B	Basal	HER2-enriched
connected nodes, miR	269	384	414	587
connected nodes, gene	2630	2731	2699	4011
edges	6942	6942	6942	6951
connected components	97	174	212	202

It should be noted that in the case of HER2, we observe a slightly higher number of edges—6951, as opposed to 6942 in the rest of the subtype networks: this is explained due to the fact that there are links that have the exact same value as the threshold for HER2, and we did not implement any tie-breaking methods; we do not consider that the presence of these marginal edges may affect our downstream analyses.

### 3.2. Identification of Commodore microRNAs: non-redundant, highly connected microRNAs

We identified 5 miRs that are non-redundant, and highly connected in at least one molecular subtype. These are:

— mir-139 and mir-150 in the Luminal A subtype
— mir-99a and mir-708 in the Luminal B subtype
— mir-136 and mir-139 in the Basal subtype.

**Figure 3.**
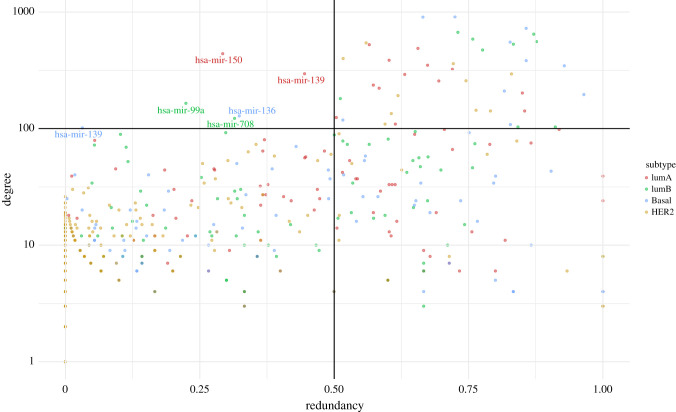
Scatter plot of degree versus redundancy coefficient for miR nodes in breast cancer molecular subtype networks. Each subtype is represented by a different colour. The plot is divided by the commodore thresholds for degree (100) and redundancy coefficient (0.5). The upper-left quadrant contains *commodore miRs*.

Figure 3 illustrates how these *commodores* are rare in the context of miRs in breast cancer subtypes. It should be noted that there are no cdre-miR in the HER2 molecular subtype, while each of the other subtypes possesses two cdre-miRs.

Another issue to highlight is the fact that mir-139 is a commodore in both the Luminal A and Luminal B subtypes. The scatter plot shows, however, that they do not exhibit the exact same behaviour in terms of connectivity and redundancy. Electronic supplementary, file 3, contains the degree and redundancy values of each cdre-miR in every subtype, which showcases that the behaviour of miRs is different in each breast cancer manifestation.

### 3.3. Functional enrichment of Commodore microRNA neighbourhoods

We analysed whether the neighbourhoods of each cdre-miR could be associated with biological functions, by means of a hypergeometric test. We found that all cdre-miRs identified are linked in this fashion to a number of biological processes, as seen in table 2. The whole set of enriched processes is found in electronic supplementary material, file 4.

**Table 2.** Enriched gene ontology biological processes in the gene neighbourhoods of cdre-miRs.

subtype	miR	enriched GO-BP
Luminal A	hsa-mir-139	113
Luminal A	hsa-mir-150	170
Luminal B	hsa-mir-708	36
Luminal B	hsa-mir-99a	46
Basal	hsa-mir-136	102
Basal	hsa-mir-139	19

**Figure 4.**
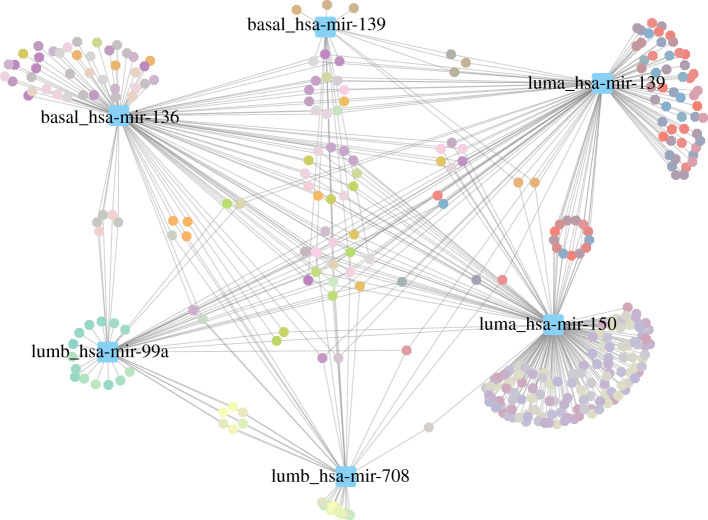
miR–gene ontology biological process network, containing all Commodore miRs found in each subtype. The colour of GO-BP nodes represents groups of functionally similar processes.

**Figure 5.**
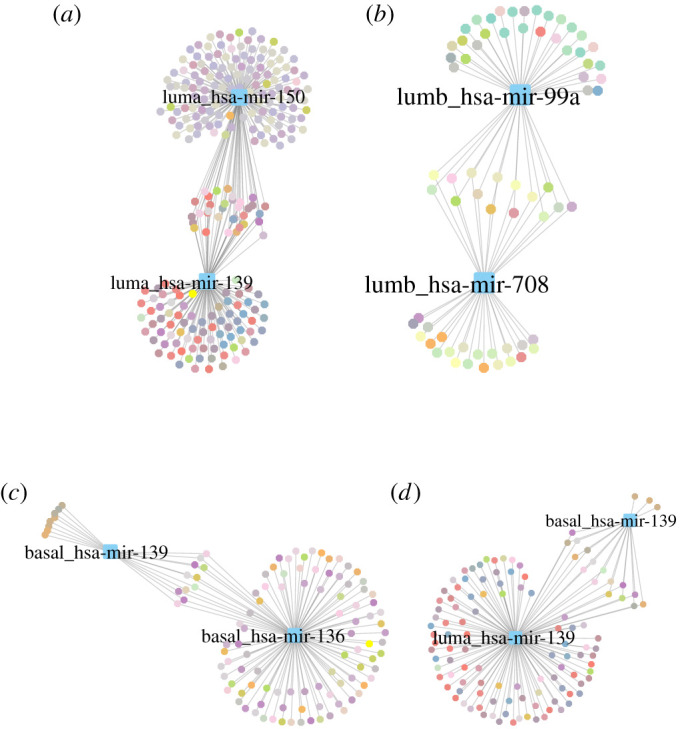
Commodore miR–gene ontology biological process for molecular subtypes (*a*–*c*) and the processes controlled by miR-139 in Luminal A and Basal subtypes. GO-BP node colours represent functionally similar processes. (*a*) Luminal A, (*b*) Luminal B, (*c*) Basal, (*d*) mir-139.

In figure 4, we represent the biological processes associated with each cdre-miR as a network. This helps illustrate how there are some processes associated with several cdre-miRs, while each cdre-miR has a set of processes that are uniquely associated with it. Since cdre-miRs are phenotype dependent, figure 5 helps illustrate more clearly the way in which cdre-miRs are associated with different functions in each subtype. Finally, in figure 5*d* the different behaviour of mir-139 in the Luminal A and Basal subtypes is illustrated.

Each panel in figure 5 shows an overlap between the functions associated with each cdre-miR: this could be due to similarity between their respective neighbourhoods. In electronic supplementary material, file 5, we provide a similarity matrix of each cdre-miR neighbourhood to show that this is not the case. In other words, each cdre-miR is affecting biological functions through different co-expressed gene sets.

#### 3.3.1. Biological processes aggregated by functional similarity

We grouped biological processes associated with each cdre-miR based on their functional similarity, as described in the methods section. The purpose of this was to reduce the number of GO-BP terms and aggregate them into the most representative (and biologically informative) terms. In table 3, we show, for demonstration purposes, the characteristic terms for mir-139 in the Luminal A subtype. The full set of groups is provided as electronic supplementary material, file 6.

**Table 3.** Luminal A, miR-139.

group number	characteristic GO-BP	characteristic GO-BP name	number of GO-BPs
1	GO:0001525	angiogenesis	14
2	GO:0007186	G protein-coupled receptor signalling pathway	15
3	GO:0010628	positive regulation of gene expression	7
4	GO:0043066	negative regulation of apoptotic process	17
5	GO:0006954	inflammatory response	15
6	GO:0006936	muscle contraction	11
7	GO:0006869	lipid transport	13
8	GO:0006069	ethanol oxidation	9
9	GO:0070374	positive regulation of ERK1 and ERK2 cascade	7
10	GO:0098609	cell–cell adhesion	5

### 3.4. Literature validation results

We systematically searched the biomedical literature to identify previous mentions of the identified biological functions associated with each cdre-miR. Table 4 shows the cdre-miR/function pairs for which at least one literature mention was found.

**Table 4.** Literature validation of biological functions associated with cdre-miRs.

subtype	miR	GO representative term	Pubmed mentions
Luminal A	hsa-mir-139	angiogenesis	3
Basal	hsa-mir-139	angiogenesis	3
Luminal B	hsa-mir-708	cell adhesion	1
Basal	hsa-mir-136	cell adhesion	5
Basal	hsa-mir-139	cell adhesion	2
Luminal A	hsa-mir-139	negative regulation of apoptotic processes	2
Luminal A	hsa-mir-139	positive regulation of gene expression	7
Basal	hsa-mir-136	regulation of signalling receptor activity	1
Luminal A	hsa-mir-150	signal transduction	30

## 4. Discussion

In previous work [23], we identified non-redundant, highly connected miRs in miR–gene co-expression networks of breast cancer. We proposed that these so-called ‘commodore’ miRs are important regulatory elements, as they are potentially able to influence the expression level of a large set of genes by themselves. Furthermore, through this regulatory action, these miRs could be able to regulate specific biological processes. As such miR behaviour was not found in healthy breast tissue networks, we speculated that cdre-miRs could confer adaptive advantages to the tumour phenotype.

In this work, we explored cdre-miRs in the context of different manifestations of breast cancer: the molecular subtypes. We compared and contrasted these cdre-miRs, as well as their associated functions, and identified common and unique traits across the breast cancer landscape. In what follows, we will be discussing key findings of our analyses; we should point out that the full set of reconstructed networks, as well as the sets of cdre-miR gene neighbourhoods, are provided as electronic supplementary material, files 7 and 8. These open datasets may lead to further insights beyond what is currently discussed in this paper.

### 4.1. Differences in microRNA roles in breast cancer subtypes

Considering that expression patterns are different between the molecular subtypes, we expected to find different sets of cdre-miRs associated with each molecular subtype. This was the case for three subtypes: Luminal A, Luminal B and Basal. In the case of the HER2-enriched, we did not find any miR that was considered a commodore as by our previously established definition.

For each of the remaining subtypes, we identified two miRs that we considered to be highly connected and non-redundant. None of the subtypes had the same pair of cdre-miR. Indeed, the only miR that had a commodore behaviour in two subtypes was mir-139, in the Luminal A and Basal subtypes; nevertheless, as we mentioned in the Results section, this miR is linked to a different gene set, as well as a different function set, in each subtype.

Previous studies have shown that the expression patterns of molecular subtypes are different not only for genes, but also for miRs [8]. Since co-expression networks can be thought to be abstractions of the regulatory program behind these expression patterns [32], it is expected to find differences in these networks between subtypes, including differences in central nodes in the network. The fact that non-redundant, highly connected miRs emerge in most (but not all) subtypes could be indicative that having such regulatory element provides an advantage for the cancer phenotype.

### 4.2. Functional roles of Commodore microRNAs

The five cdre-miRs found with our methodology have been previously reported to be determinant in breast cancer, as well as other types of cancer. Such is the case of miR-139. This miR has been found to be a regulator of metastasis-related pathways in breast cancer [33]. miR-139 has been also reported as a suppressor of invasion and migration in breast cancer cell lines, by targeting RAB1A gene [34]. Additionally, it controls resistance to radiotherapy by targeting pathways of DNA repair [35]. This miR has been observed in colorectal and gastric cancer [36,37]. In fact, miR-139 is considered as a biomarker for gastric cancer. It is important to mention that for breast cancer and breast cancer cell lines, this miR acts on luminal or basal-like cell lines [33,34], which coincides with our finding of miR-139 as a cdre-miR in Luminal A and Basal breast cancer subtypes.

In the case of miR-150, it has been observed to suppress metastasis in triple-negative breast cancer by targeting HMGA2 gene [38]. However, it has been reported to have an opposite behaviour. miR-150 promotes growing and invasion in breast cancer cell lines by targeting the P2X7 receptor, which is a pro-apoptotic protein [39]. Further investigation is necessary to clearly distinguish the dual behaviour of this molecule.

The case of miR-99 and miR-708 (cdre-miRs for Luminal B subtype) is interesting, since there are no reports of its effect in Luminal B breast cancer subtype or Luminal B-like cell line. In fact, in Luminal A subtype the miR cluster mir-99a/let-7c/mir-125b-2 is upregulated compared with Luminal B [40]. However, it has been reported that miR-99a reduced breast cancer cell proliferation, invasion and migration by targeting FGFR3 [41]. In a similar case, miR-708 is considered a possible potential target in triple-negative breast cancer, since miR-708 targets inhibit proliferation pathways in MCF7 and MDA-MB-231 breast cancer cell lines [42], and reduce metastasis in triple-negative breast cancer [43].

In the Basal subtype, we found two cdre-miRs, miR-136 and the already mentioned miR-139. miR-136 is considered a potential target for cancer therapy since it suppresses invasion and metastasis [44,45]. In 2019, Tang *et al*. [46] observed that miR-136, miR-139-3p, mir-139-5p, and others resulted underexpressed in triple-negative breast cancer. This report strongly supports our finding regarding the functional relevance of miR-136 and miR-139 in Basal-like breast cancer subtype.

All the aforementioned reports regarding the crucial role that those miRs exert in cancer phenotypes reinforces our hypothesis of that miRs with low redundancy, but a high number of targets (high node degree) may serve as potential targets for a directed therapy. Further investigation is necessary; however, this approach opens the possibility that an automated bioinformatic pipeline may suggest novel potential biomarkers for other types of cancer.

### 4.3. Possible advantages of studying Commodore microRNAs in breast cancer

It is well known that miRs provide a regulatory mechanism for the control of gene expression [47]. Also known is the fact that miRs are widely deregulated in most cancers, although whether these are at the genesis of the disease, or a consequence of the pathological state, it is not known [48]. Since cancer is a complex disease, it is possible that both situations could happen, and even coexist.

Potentially oncogenic miRs are able to confer functional features to cancer through their action as regulatory elements of gene expression [49]. Highly central miR nodes in miR–gene co-expression networks could act as control elements of gene expression based on their network connectivity, just like other genetic elements have been identified [50]. By controlling the expression of genes involved in biological functions, these miRs could in turn control the activity of the function itself. In this context, cdre-miRs, both highly connected and non-redundant, could theoretically be the primary drivers of specific alterations of biological function.

### 4.4. Functional heterogeneity and functional convergence

Having different cdre-miRs in each subtype leads to a varied landscape of altered functions. As we have shown, each cdre-miR in each subtype is associated with the expression of different genes, which in turn leads to differences in the associated functions. We observe that each cdre-miR has a set of functions that are unique to it, in the context of the phenotype in which it acts as a commodore. This could be one of the origins of the functional diversity observed and widely reported in breast cancer [51].

On the other hand, we observe that some functions may be affected by different cdre-miRs, either in the same or in different subtypes. The first explanation for this could be related to the (small) overlaps in gene neighbourhoods observed between the cdre-miRs. But on a deeper level, this could be indicative of a convergence in biological process (de-)regulation. In other words, the control of a given function (or a significant subset of said function) may confer an advantage to the tumour phenotype, which emerges regardless of the clinical (or molecular) manifestation, through different regulatory mechanisms. This could also be related to the lack of cdre-miRs in the HER2-enriched molecular subtype: being mostly driven by the amplification of a genomic region [52], the emergence of cdre-miRs is not needed for the development of this disease manifestation.

When we observe the terms that define the groups of functions associated with our cdre-miRs, it can be observed that several of these refer to well-known processes altered in cancer. Furthermore, when we look at the list of processes that were previously mentioned in the literature as being associated with breast cancer, we see that all of these belong to the set of functions known as the *hallmarks of cancer* [53,54]. While experimental validation is still needed, if cdre-miRs are indeed acting as functional control elements specific to different breast cancer manifestations, then these could be attractive therapeutic options in the context of precision medicine [55–57].

### 4.5. Limitations and future directions

As with other data-driven approaches to understand transcriptional alterations in cancer, there are some considerations to be made to reach meaningful conclusions. The presented results depend on (i) how reliable the data generation process is; (ii) the use of a proper data preprocessing pipeline; (iii) the suitability of the downstream analysis pipeline. In this regard, we relied on the well-documented and widely validated processes used by the Cancer Genome Atlas for data generation and preprocessing. The downstream analysis pipeline presented here, on the other hand, builds on work developed by many groups, including ours, around network reconstruction using information-theoretic measures [5,7,14,23,32] and the wider computational biology community [58–60].

One open issue remains the way to properly validate the results. The most straightforward validation is perhaps the use of a secondary validation dataset. In this regard, previous studies by our group [61] have used the METABRIC dataset [62]. However, for the purpose of network reconstruction, issues such as batch effects and sample sizes may introduce additional confounding factors. An alternative approach is the use of cross-validation strategies, such as the ones used for recent multiomic network reconstructions [63]. Such approaches have shown that these methods lead to robust link prediction results.

For this particular work, our focus is to validate not only the predictions, but whether action on these miRs offers control on the identified biological activities. For that purpose, an experimental approach is needed. By using a combination of *agomirs*, *antagomirs*, electrophysiology and biochemical measures, quantitative results on the effect of our proposed miRs will be gathered.

## 5. Conclusion

In this work, we identify highly connected, non-redundant cdre-miRs in the context of breast cancer molecular subtypes. cdre-miRs may become important regulatory elements whose functionality arises from their hierarchy in the co-expression networks. We found that different molecular subtypes exhibit different sets of these cdre-miRs, each associated with a specific set of biological functions. A number of these functions are relevant for the tumour phenotype. Such is the case of angiogenesis, cell adhesion, regulation of apoptosis and regulation of inter- and intracellular signalling. We observed that some of the associated functions are unique to each subtype, reflecting their functional diversity, while others are common. In many cases, these functions may be behind robustness of the tumour phenotype via adaptive processes. We found evidence in the literature that supports the fact that some of these functions are indeed affected by our set of identified miRs. Such functions are, as stated, well-known hallmarks of cancer, which could make targeting these miRs a potential therapeutic alternative for different breast cancer manifestations. Detailed functional studies both *in vitro* and *in vivo* are needed, however, in order to pave the way to clinical interventions based on this small, specific set of molecular targets.

## Data accessibility

Data used were obtained from the Cancer Genome Atlas through the Genome Data Commons: https://gdc.cancer.gov/. The list of URLs for each individual dataset, as well as all other code needed to reproduce the results of this paper can be found at https://github.com/guillermodeandajauregui/cdre-miR-BrCanSub.
